# Global genomic methylation related to the degree of parasitism in cattle

**DOI:** 10.1038/s41598-022-22753-5

**Published:** 2022-10-28

**Authors:** Ricardo Velludo Gomes de Soutello, Maria Gabriela Fontanetti Rodrigues, Juliana Alencar Gonçalves, Hornblenda Joaquina Silva Bello, Bruno Ettore Pavan, Ester Silveira Ramos

**Affiliations:** 1 Faculty of Agricultural and Technological Sciences (FCAT), São Paulo State University “Júlio de Mesquita Filho” (UNESP), Comandante João Ribeiro de Barros Highway, km 651-Bairro das Antas, Dracena, SP 17900-000 Brazil; 2 School of Veterinary Medicine and Animal Science (FMVZ), São Paulo State University “Júlio de Mesquita Filho” (UNESP), Prof. Doutor Walter Mauricio Correa Street, s/n, Botucatu, SP 18618-681 Brazil; 3 Faculty of Engineering (FEIS), São Paulo State University “Júlio de Mesquita Filho” (UNESP), Brasil Sul Avenue, 56, Ilha Solteira, SP 15385-000 Brazil; 4 Ribeirão Preto Medical School (FMRP), University of São Paulo (USP), Bandeirantes Avenue, 3900, Monte Alegre, Ribeirão Preto, SP 14049-900 Brazil

**Keywords:** Biotechnology, Genetics, Immunology

## Abstract

The objective of the present study was to characterize a herd of 72 ½ Angus × ½ Nellore heifers, identify the resistant, resilient and susceptible animals to parasites, relate the overall DNA methylation of these animals with the degree of parasitism, evaluated by the egg count per gram of feces (EPG), *Haematobia irritans* count (horn fly) and *Rhipicephalus microplus* count (bovine tick). The experiment was carried out in a completely randomized design, containing 72 treatments, with each animal considered a treatment, and 11 repetitions, with each collection within a year considered a repetition. The data obtained from the counts of the evaluated parasites were subjected to statistical analysis using the SISVAR program, to classify heifers according to the degree of parasitism in low (resistant), intermediary (resilient) and high (susceptible) parasite load for infection by nematodes, infestation by ticks and flies. Addition the animals in these three groups, by hierarchical grouping using the GENES program, heifers were classified as to the degree of parasitism by the three parasites along with the DNA methylation content of the animals in each group. A negative relationship was observed between resistance and methylated DNA content in both classifications, with the resistant, resilient, and susceptible animals showing the highest, intermediate, and lowest methylated DNA quantifications, respectively. Thus, the methodologies used herein enabled the classification of 72 heifers according to the degree of collective infection by gastrointestinal nematodes and infestation by ticks and horn flies, thereby establishing a link between the degree of parasitic resistance in cattle and the global methylated DNA quantification.

## Introduction

Parasitic diseases constitute a major cause of reduced productivity in ruminants^1^, with *Rhipicephalus microplus* (cattle tick), *Haematobia irritans* (horn fly), and gastrointestinal nematodes being the main parasites^2^. Their control is, thus, extremely important and is performed mainly via the use of antiparasitic drugs (anthelmintics, acaricides, and insecticides)^3^. However, the indiscriminate and frequent use of the same pharmacological agents leads to the emergence of antiparasitic resistance^4^, thus necessitating a search for parasite control alternatives, such as the use of resistant animals.

The mechanisms involved in the animal resistance process are complex and poorly understood. It is known that resistance is a heritable trait that depends on a large number of antibodies, cytokines, cells, and genes^5,6^, with a role in the expression of molecules that regulate the host's immunity, that is, molecules that regulate the response capacity to antigens, in this case, antigens found in parasites. This response consists of the clustering, targeting and activation of leukocytes, mainly eosinophils^7,8^, which are indicators of resistance against parasites and can be found in blood samples. However, the establishment and maintenance of phenotypes are independent of the genetic material and occur via epigenetic marks or mechanisms that modify gene expression^9^; such phenotypes to transgenerational effects are reversible and also heritable^10^.

DNA methylation is one of the most studied epigenetic mechanisms, involving the addition of a methyl radical (-CH3) at the carbon-5 position of a cytosine, yielding 5-methylcytosine (5mC)^11^. It normally occurs in DNA sequences with a high density of cytosine and guanine bases (CpG islands)^12^ and its location in a gene can either silence, activate, or overexpress the gene^13^.

Thus, research on epigenetic mechanisms can be used as a tool for the identification and possible selection of resistant animals. Studies in this field, correlating the methylated content in bovine DNA and parasitism, are non-existent. Thus, the present study aimed to characterize a herd of 72 ½ Angus × ½ Nellore heifers evaluated during an one-year period without anti-parasitic treatment, to identify the animals resistant, resilient, and susceptible to endo and ectoparasites by correlating the global DNA methylation of these animals with the degree of parasitism.

## Material and methods

The experiment was conducted in accordance with the ethical guidelines for experimentation on animals of the Ethics and Animal Welfare Committee of the Faculty of Agricultural and Technological Sciences of São Paulo State University “Júlio de Mesquita Filho” (UNESP), Dracena (FCAT/UNESP). The study was approved by the ETHICS COMMISSION IN THE USE OF ANIMALS—CEUA of the College of Agricultural and Technological at UNESP under registration number 13/2017.

Seventy-two first-cross ½ Angus × ½ Nellore heifers from the same homogeneous herd of fixed-time artificially inseminated Nellore cows using semen from a single Aberdeen Angus bull, were used. This crossbreed is currently the most used in Brazil, given the great demand for this type of animal due to its precocity, carcass quality and adaptation to the country's climatic conditions^14^.

The heifers remained in the same environment from birth to the end of the study, as did their dams, which had the same genetic characteristics and were kept in the same batch and under the same conditions throughout pregnancy and lactation. The animals only received antiparasitic treatment two months prior to the beginning of the evaluations, which started at weaning at 8 months of age, comprising levamisole 18.8% injected subcutaneously at a dose of 4.7 mg/kg of body weight for the control of gastrointestinal helminths, and cypermethrin (7 mg/kg), applied on the dorsal line, for the control of ectoparasites; no further anti-parasitic treatment was administered over the following year.

The design used was completely randomized, considering each animal as a treatment (72 treatments) with 11 repetitions, referring to the number of collections performed. Afterwards, the counts of eggs per gram of feces (EPG), numbers of ticks and numbers of horn flies were submitted to statistical analysis to divide the heifers into three groups by statistical difference (p < 0.05): resistant to parasites, resilient and susceptible.

Resistant animals are those that manage to eliminate or suppress the development of parasites, without presenting a parasitic or very low load. Susceptible animals are animals with a high parasitic load, whose performance is impaired by parasites. Resilient animals are those that have a parasitic burden however they suffer little damage due to the parasites^5^.

The degree of parasitism was evaluated through fecal collection and horn fly and tick counts, every 28 days. Feces were collected directly from the rectum of the animals for coprological exams, conducted at the Laboratory of Parasitology and Animal Health in the Faculty of Agricultural and Technological Sciences, by counting eggs per gram of feces (EPG) using a McMaster chamber^15^. Coproculture and larvae extraction were also performed^16^ for later identification of the genera present therein^17^.

The counting of horn flies entailed leading the heifers into the corral for quantification of their cervical-dorsal-lumbar region during the mildest periods of the day (the morning)^18^. The evaluation of ticks was performed by quantifying engorged females with a size equal to or greater than 4.5 mm on one side of the animal and then multiplying by two^19^.

### Global DNA extraction and methylation

Total genomic DNA was extracted from the blood, collected in the last evaluation, using the EasyPureBlood Genomic DNA Kit (Transgen Biotch, Beijing, China). Sample quantification and quality assessment were performed using a spectrophotometer (NanoDrop2000—Thermo Scientific).

Methylation analysis was performed using the Imprint DNA Methylation Quantification kit (Sigma), using strips with pre-treated wells containing methylated DNA-binding reagent and using a DNA methylation-sensitive capture antibody and a detection antibody, allowing colorimetric detection of the absolute amount of DNA methylation in each animal. The absorbance of the solution contained in the wells was measured on an ELISA spectrophotometer (Kasuaki-DR-200Bs-BI) at a wavelength of 450 nm.

### Statistical analysis

The distributions of EPG, tick, and horn fly counts were verified through the Shapiro–Wilk test using SISVAR 5.4^20^.

The EPG, tick, and horn fly counts were analyzed separately in a completely randomized design, comprising 72 treatments and 11 replicates for each collection; the means of each variable were subjected to analysis of variance (ANOVA) and subsequent statistical clustering analysis by the Scott–Knott test (5%) using SISVAR 5.4^20^. Thus, the animals in the herd were, according to the degree of parasitism based on the statistical difference of the means of each animal, clustered into three categories for each variable: resistant, resilient, or susceptible.

The general classification based on the degree of parasitism was performed using Selegen software^21^, and a dendrogram was subsequently created using the hierarchical clustering method (Ward’s method) of the 72 heifers considering the three variables simultaneously (EPG, tick, and horn fly counts); the genetic distances were calculated^22^. Thus, the animals were also classified into three groups: resistant, resilient, and susceptible, and the average counts of the three variables were analyzed by ANOVA using SISVAR 5.4^20^, and subsequently, the means were compared by the Tukey’s test (p < 0.05). This classification was used because the animals are affected by the three parasites simultaneously in the environment, so the resistance mechanisms occur for the three parasites.

The statistical analysis of global DNA methylation was performed in a completely randomized design, with three treatments (resistant, resilient, or susceptible) and 72 repetitions, using the absolute absorbance values and the mean values of methylation difference between animals transformed as $\sqrt[2]{n+0.5}$, where n is absolute absorbance values of methylation, the which were analyzed by ANOVA using SISVAR 5.4^20^ to evaluate the statistical significance between the different methylation values of the three groups; the counts of gastrointestinal helminths, ticks, and horn flies were analyzed by the Tukey’s test (5%).

With the study it was possible to evaluate two different forms of classification through statistical analysis, the first classifying the animals using the SISVAR program according to the degree of parasitism caused by helminths, ticks and horn flies separately, and the second form classifying the animals through the GENES program through a hierarchical grouping that considered the degree of parasitism by helminths, ticks and flies, correlating them with DNA methylation, simultaneously.

### Compliance with ethical standards

The authors assert that all procedures contributing to this work comply with the ethical standards of the Ethics and Animal Welfare Committee of the Faculty of Agricultural and Technological Sciences of São Paulo State University “Júlio de Mesquita Filho”, Dracena, SP (FCAT/UNESP), approved under registration number 13/2017. All animal experiments were adhered to ARRIVE guidelines.

## Results

**Figure 1:**
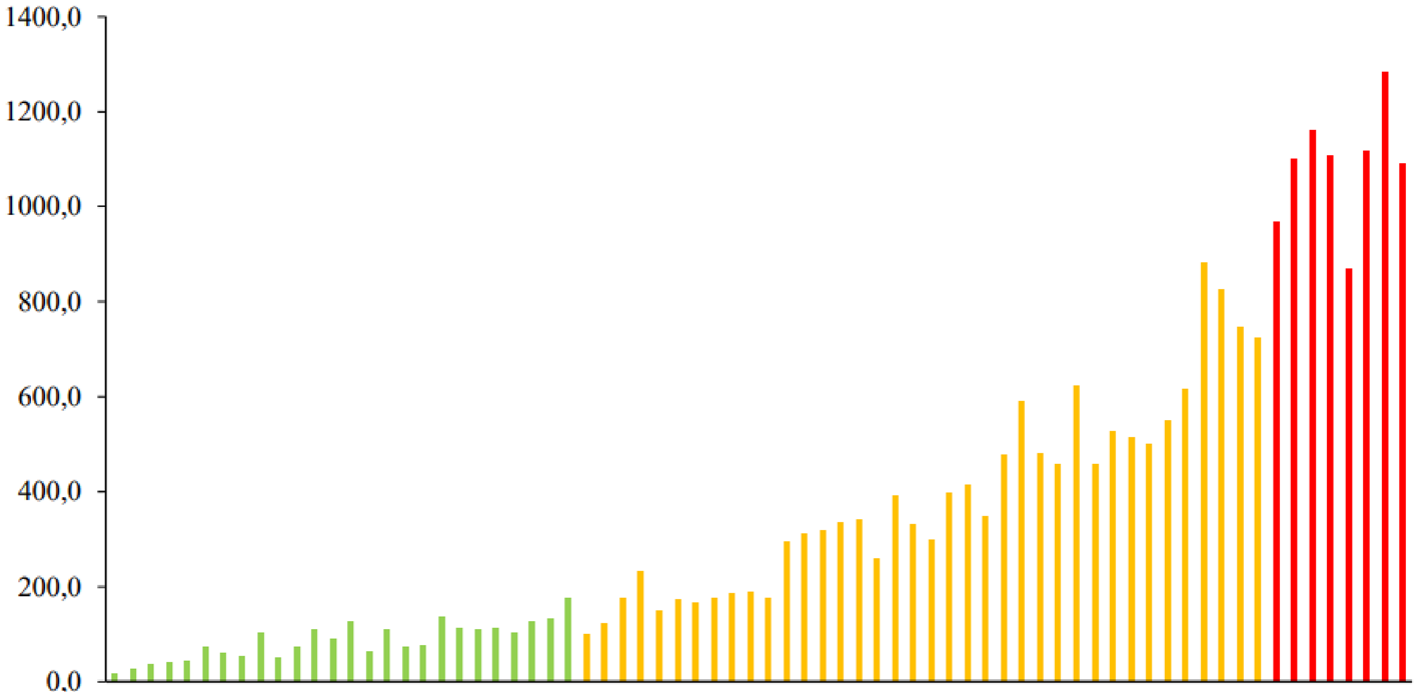
Distribution of 72 ½ Angus × ½ Nellore heifers based on number of eggs per gram of feces (EPG) counts. Green, yellow and red bars means resistant, resilient and susceptible animals, respectively.

**Figure 2:**
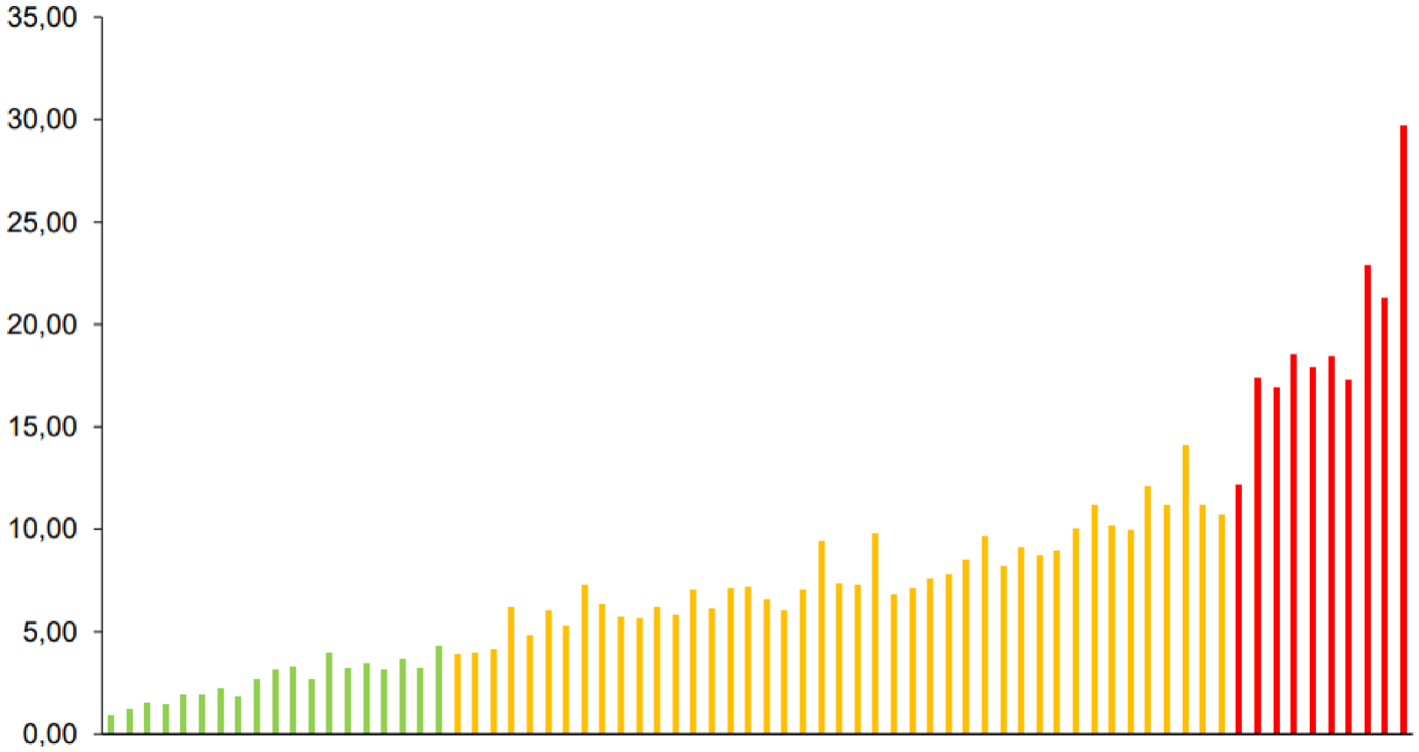
Distribution of 72 ½ Angus × ½ Nellore heifers based on tick counts. Green, yellow and red bars means resistant, resilient and susceptible animals, respectively.

**Figure 3:**
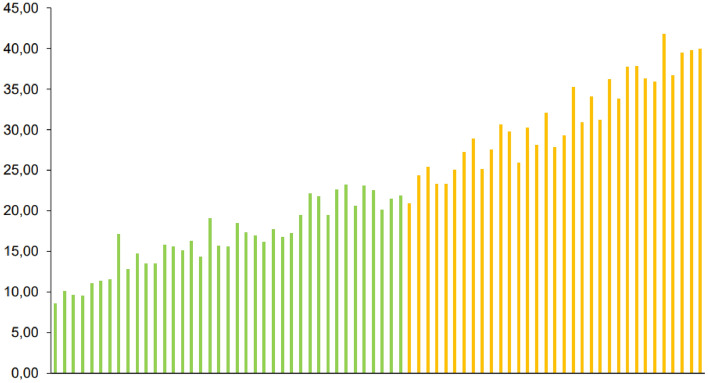
Distribution of 72 ½ Angus × ½ Nellore heifers based on horn fly counts. Green, yellow means resistant and resilient animals, respectively.

The individual EPG, tick and horn fly counts of the 72 heifers showed significant differences between the animals (*p* < 0.05), enabling the distribution of the herd into three categories: resistant, resilient, or susceptible to infections by gastrointestinal nematodes, infestation by *R. microplus* and infestation by *H. irritans,* respectively (Figs. 1, 2 and 3).

Table 1 shows the mean EPG, tick and horn fly counts values, the global DNA methylation content (absolute absorbance value), and the number of animals per group of 72 heifers classified as resistant, resilient, or susceptible. There were significant differences (*p* < 0.05) between the average variables counts of the animals per classification category.

**Table 1:** Average number of eggs per gram of feces (EPG), *Rhipicephalus microplus* (cattle tick), *Haematobia irritans* (horn fly), global DNA methylation quantification (absolute absorbance value), and number of heifers per group (N) distributed into resistant, resilient, and susceptible groups, by Scott-Knott test (5%).

Parasite	Classification	N	(%)	Average	Methylation DNA
EPG gastrointestinal nematode	Resistant	26	36	86 a*	0.238
	Resilient	38	53	392 b	0.225
	Susceptible	8	11	1087 c	0.197
	Mean			359	0.227
	CV (%)	74.65		15.19	9.32
Cattle tick	Resistant	19	26	3 a	0.295 a
	Resilient	43	60	8 b	0.216 b
	Susceptible	10	14	19 c	0.151 c
	Mean			8	0.227
	CV (%)	50.42		9.61	8.46
Horn fly	Resistant	39	54	17 a	0.245
	Resilient	33	46	32 b	0.205
	Mean			23	0.227
	CV (%)	40.72		9.55	11.15

*Different letters in the column show significant differences between groups by the Scott-Knott’s test (*p* < 0.05).

For infections by gastrointestinal nematodes, the genera of helminths found through co-culture showed a higher prevalence of *Haemonchus* spp. (44.47%) and *Cooperia* spp. (30.17%), followed by *Oesophagostomum* spp. (15.5%) and *Trichostrongylus* spp. (9.86%).

The frequency distribution of EPG counts in heifers throughout the evaluation period, which included a total of 792 evaluations, showed that 47% (373) of the evaluations exhibited a low EPG count (0–50), whereas only 12.5% (99) showed high counts (800–4000 EPG, or over 4000 EPG).

One can observe that there were no significant differences among the global DNA methylation contents of the three groups (Table 1).

For infestation by *R. microplus*, the frequency distribution of tick counts in the 72 heifers throughout the evaluation period showed that 98% (778) of the evaluations performed yielded low numbers (0–50 ticks); only 2% (14) showed counts of over 50 ticks.

Unlike the case of the infection by helminths, there were significant differences among the global DNA methylation contents of the three groups (*p* < 0.05) (Table 1).

For infestation by *H. irritans,* the frequency distribution of the fly counts in the 72 heifers, throughout the evaluation period (792 evaluations in total), showed that 87% (689 examinations) had a count between 0 and 50 flies, 11% (89 examinations) between 50 and 100 flies, and only 2% (14 examinations) above 100 flies/animal.

In this case, there was no significant difference between the global DNA methylation content of the two groups; however the link between the mean horn fly count values and the methylated DNA quantifications of the animals can be observed; the group of resistant animals exhibited the highest methylated DNA quantification, whereas susceptible animals exhibited the lowest.

### General classification by degree of parasitism and global DNA methylation content

**Figure 4:**
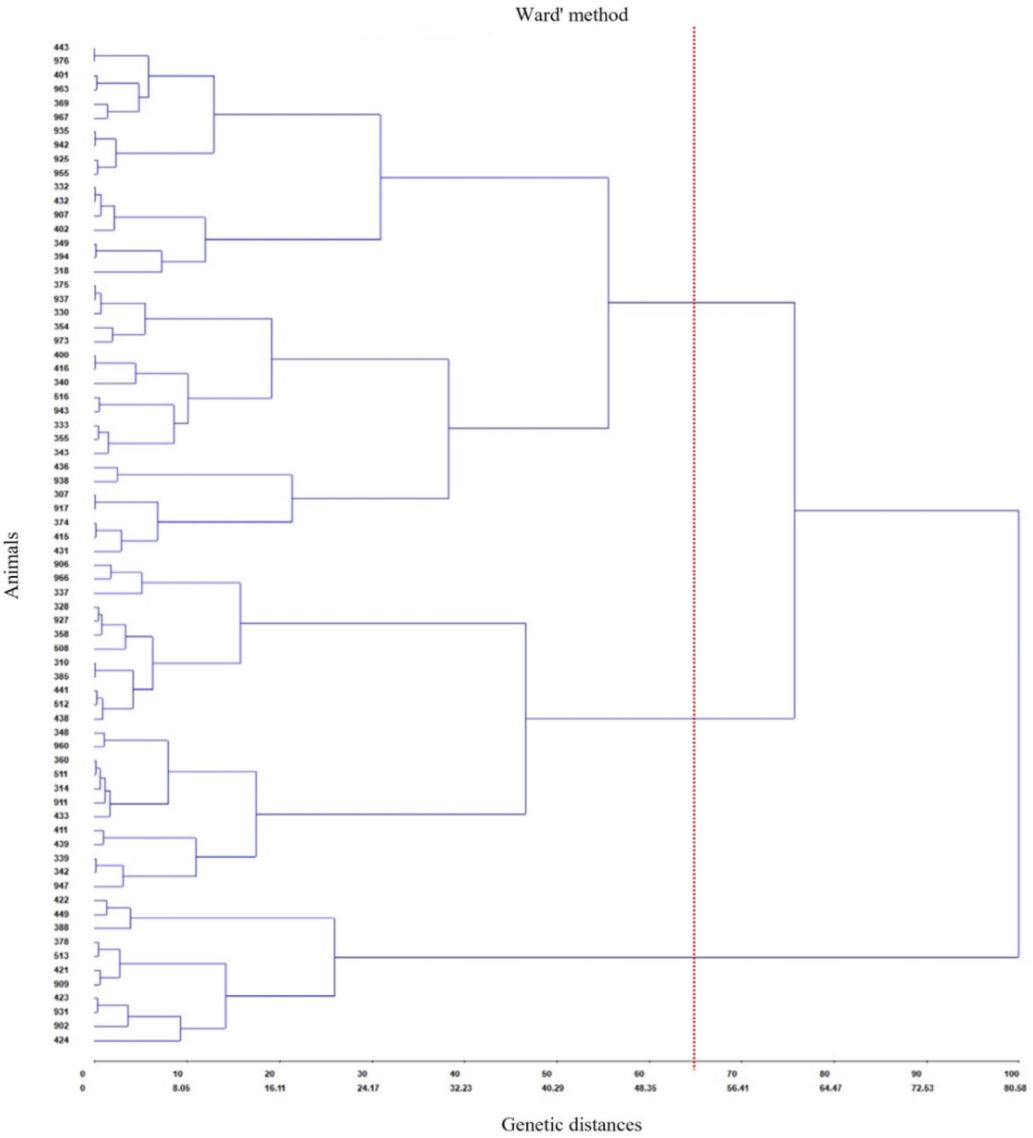
Dendrogram of the clustering of 72 ½ Angus × ½ Nellore heifers based on three traits (number of eggs per gram of feces (EPG) and tick and horn fly counts), studied using Ward's method. Red line indicates the cut used to general classification.

Figure 4 shows the hierarchical clustering of the 72 heifers according to parasitism resistance to gastrointestinal helminths, ticks, and horn flies calculated with GENES using Ward's method. It was possible to classify heifers into three groups: parasite-resistant (33.3%), -resilient (51.4%), or -susceptible (15.3%) animals.

Table 2 shows the mean EPG, tick, and horn fly counts; the global DNA methylation content (absolute absorbance value); and the number of animals per group of the 72 heifers classified as either resistant, resilient, or susceptible. The EPG counts were significantly different among the three groups (*p* < 0.001), whereas *R. microplus* and *H. irritans* counts differed significantly (*p* < 0.001) only between the resistant and the resilient and susceptible groups; the latter two did not differ significantly from one another.

**Table 2:** Average number of eggs per gram of feces (EPG), *Rhipicephalus microplus* (cattle tick), *Haematobia irritans* (horn fly), global DNA methylation quantification (absolute absorbance value), and number of heifers per group (N) distributed into resistant, resilient, and susceptible groups, by GENES program.

Classification	N	EPG	Cattle tick	Horn fly	Methylation DNA
Resistant	24	307 a*	3.4 a	19 a	0.247 a
Resilient	37	205 b	10.4 b	26 b	0.227 ab
Susceptible	11	988 c	10.2 b	24 b	0.185 b
Mean		359	8	23	0.227
CV (%)		23.92	19.11	14.29	63.84

*Different letters in the column show significant differences between groups by the Tukey’s test (*p* < 0.05).

The global DNA methylation quantification showed significant differences between resistant and susceptible animals (*p* < 0.05) (Table 2), thus allowing the establishment of a link between parasitic resistance in animals and global DNA methylation content, with the resistant animals exhibiting greater methylated DNA quantifications, whereas susceptible animals exhibited lower contents. The resilient animals group exhibited an intermediate global methylation content, which did not differ significantly from the others.

## Discussion

The classification of heifers according to the degree of infection by gastrointestinal nematodes (Table 1) was in line with the literature, that described a 25:50:25 resistant:resilient:susceptible animal ratio, with the average EPG counts of the animals in each category being typical of infections by gastrointestinal nematodes^5^. In other work, mean counts under 200 EPG represent a mild degree of infection; between 200 and 700 EPG represent a moderate degree; and above 700 EPG represent a high degree of infection^23^. It can also be observed that the mean EPG counts in resistant animals were close to that in Nellore cattle, that observed EPG counts below 50 for resistant animals^24^.

Regarding the distribution of heifers per degree of tick infestation (Table 1), different methods of cattle classification can be found in the literature.^25^, for example, classified 2 Canchim female cattle groups according to the total number of ticks counted in each animal. It was reported that 87.5% and 12.5% of an Angus × Nellore heifer herd were resistant (14 animals) or resilient (2 animals), respectively^26^.

In the present study, the tick counts in all heifers, including those classified as susceptible, were average. It is possible to observe that the average tick counts (Table 1) were close to the counts observed in zebu animals or crossbreds with a higher percentage of zebu blood^27,28^. It was reported mean tick counts (transformed data) of 3.47 (Nelore), 10.22 (Camchim × Nellore), 17.2 (Angus × Nellore) and 26 (Simental × Nellore)^27^, and it was observed counts averages of 8.52 ticks/animal (Nelore), 18.81 ticks/animal (Nelore × Senepol) and 75.34 ticks/animal (Nelore × Angus)^28^.

In a study using also using heifers Angus × Nellore, showed tick counts reporting loads from 0 to 100 ticks/animal for most animals (68.75%), with 87.5% classified as resistant and 12.5% as resilient^26^.

For the infestation by *H. irritans*, heifers were classified into only two categories (resistant or resilient) (Table 1), differing from the distribution using two clustering methods—descriptive analysis and best linear unbiased predictions (BLUPs)—and reported that the former identified 16% and 10.7% of the animals as resistant and susceptible, respectively, whereas the latter clustered 12% of the animals as resistant and 12% as susceptible^29^.

The mean horn fly counts for both categories (Table 1) were within the tolerable range, being below the 50–300 flies/animal considered harmful for cattle^18^. These counts, as well as the general average fly count (Table 1) were also similar to the infestation observed for crossbreed *Bos indicus* cattle. It was observed an infestation of 17 flies/animal for Angus × Nellore cattle, a value closer to that found in the present study^27^.

The frequency distribution of horn fly counts in heifers is similar to that found in the literature for Nellore animals, which observed 50% of animals with infestations below 50 flies/bovine, 38% between 50 and 100 flies, 10% between 100 and 150 flies, and only 2% with more than 150 flies/bovine^30^.

GENES clustered the 72 heifers into three categories (resistant, resilient, or susceptible) by the hierarchical clustering of the animals (Fig. 4), according to parasitism resistance to gastrointestinal nematodes, *R. microplus*, and *H. irritans*. The distribution found in the present study (Table 2) is similar to the 25:50:25 ratio suggested for the EPG count, where the majority of a herd comprises resilient animals^5^. EPG, tick, and horn fly counts, described in Table 2, also showed ratios and statistical differences similar to those found for each separate variable in the classification analyzed by the Scott–Knott (5%) and Tukey (5%) tests (Table 1).

Analyzing the global DNA methylation content (Table 2), a relationship was observed between this parameter and the degree of parasitism (gastrointestinal nematodes, ticks, and horn flies), being this the first report in the literature relating parasitic resistance and global DNA methylation in cattle. It was reported a lower DNA methylation rate in cows with high milk production and the inverse in animals with low milk production^31^. However, there is a need for further studies to verify the different patterns of DNA methylation in different types of blood cells, as methylation is influenced by cell and tissue type, such as immune system cells^32^; age; exposure to environmental stimuli; and a myriad of other factors^33,34^.

The results found in the present study provide promising information regarding the possibility that global DNA methylation in the blood is linked to the regulation of bovine resistance to parasites-involved gene expression. This fact can also be observed in studies involving cattle in other research areas^35,36^. Thus, the best way to classify animals will depend on the characteristic you want to observe, whether it is a specific parasite or whether you want to evaluate the main parasites of cattle in general.

Given the importance and the increase in the number of studies aimed at understanding epigenetic mechanisms and their relationship with cattle development, the present study is the first to evaluate the relationship between an epigenetic mechanism (global DNA methylation) and the resistance of cattle to helminths (gastrointestinal nematodes), *R. microplus* and *H. irritans*. Thus, future studies on the subject are extremely necessary to gain a deeper understanding of the factors involved in parasitic resistance.

It was possible to relate the degree of resistance in animals to parasites using the global DNA methylation content. These results are promising and encourage further studies on the subject while raising expectations regarding epigenetic mechanisms becoming a tool for the selection of parasite-resistant animals.

## Data Availability

The datasets used and/or analysed during the current study available from the corresponding author on reasonable request.

## References

[1] Oliveira PA, Ruas JL, Riet-Correa F, Coelho ACB, Santos BL, Marcolongo-Pereira C, Sallis ESV, Schild AL, Oliveira PA, Ruas JL, RieT-Correa F, Coelho ACB, Santos BL, Sallis ESV, Schild AL (2017). Doenças parasitárias em bovinos e ovinos no sul do Brasil: Frequência e estimativa de perdas econômicas. Pesq. Vet. Bras..

[2] Amarante AFT, Amarante MGV (2016). Advances in the diagnosis of the gastrointestinal nematode infections in ruminants. Braz. J. Vet. Res. Anim. Sci..

[3] Brito LG, Barbieri FS, Rocha RB, Santos APL, Silva RR, Ribeiro ES, Guerrero F, Foil L, Oliveira MCS (2019). Pyrethroid and organophosphate pesticide resistance in field populations of horn fly in Brazil. Med. Vet. Entomol..

[4] McVeigh P (2020). Post-genomic progress in helminth parasitology. Parasitology.

[5] Gasbarre LC, Leighton EA, Sonstegard T (2001). Role of the bovine immune system and genome in resistance to gastrointestinal nematodes. Vet. Parasitol..

[6] Shyma KP, Gupta JP, Singh V (2015). Breeding strategies for tick resistance in tropical cattle: A sustainable approach for tick control. J. Parasit. Dis..

[7] Brossard M, Wikel SK (2004). Tick immunobiology. Parasitology.

[8] Sykes AR (2008). Manipulating host immunityto improve nematode parasite control: *Quo vadit*. Parasite Immunol..

[9] Nora, M.A.A., Connor, T. & Ishwarlal, J. *Genetics, Epigenetic Mechanism* (StatPearls Publishing, 2021). https://www.ncbi.nlm.nih.gov/books/NBK532999. 30422591

[10] Bošković A, Rando OJ (2018). Transgenerational epigenetic inheritance. Annu. Rev. Genet..

[11] Matsuda S, Yasukawa T, Sakaguchi Y, Ichiyanagi K, Unoki M, Gotoh K, Fukuda K, Sasaki H, Suzuki T, Kang D (2018). Accurate estimation of 5-methylcytosine in mammalian mitochondrial DNA. Sci. Rep..

[12] Moore LD, Le T, Fan G (2013). DNA methylation and its basic function. Neuropsychopharmacology.

[13] Wang P, Xia H, Zhang Y, Zhao S, Zhao C, Hou L, Li C, Li A, Ma C, Wang X (2015). Genome-wide high-resolution mapping of DNA methylation identifies epigenetic variation across embryo and endosperm in maize (*Zea mays*). BMC Genom..

[14] Anualpec. In *Anuário da Pecuária Brasileira*. São Paulo: FNP Consultoria/Agros Comunicação, p. 35. (2021).

[15] Rashid MH, Stevenson MA, Waenga S, Mirams G, Campbell AJD, Vaughan JL, Jabbar A (2018). Comparison of McMaster and FECPAKG2 methods for counting nematode eggs in the faeces of alpacas. Parasites Vectors.

[16] Robert FHS, O’Sullivan PJ (1950). Methods for eggs counts and larval cultures for Strongyles infecting the gastrointestinal tract of cattle. Aust. J. Agric. Res..

[17] Keith RK (1953). The differentiation of the infective larvae of some common nematode parasites of cattle. Aust. J. Zool..

[18] Almeida, F.A., Basso, F.C., Zocoller-Seno, M.C. & Valério, W.V.F. In *Population Dynamics of the Horn Fly (Haematobia irritans) on Guzera Cattle Breed and Crossbred in Selvíria, MS*. Semina: Ciências Agrárias. 10.5433/1679-0359.2010v31n1p157 (2010).

[19] Wharton RH, Utech KBW (1970). The relation between engorgement and dropping of *Boophilus microplus* (Canestrini) (Ixodidae) to the assessment of thick numbers on cattle. Aust. J. Entomol..

[20] Ferreira DF (2014). Sisvar: A Guide for its Bootstrap procedures in multiple comparisons. Ciênc. Agrotec..

[21] Cruz CD (2016). Genes Software—extended and integrated with the R, Matlab and Selegen. Acta. Sci. Agron..

[22] Mahalanobis PC (1930). On test and measures of group divergence: Theoretical formulae. J. Asiat. Soc. Bengal.

[23] Ueno, H. & Gonçalves, P.C. *Manual Para Diagnóstico das Helmintoses de Ruminantes* (Japan International Cooperation Agency, 1998).

[24] Bricarello PA, Zaros LG, Coutinho LL, Rocha RA, Kooyman FNJ, De Vries E, Gonçalves JRS, Lima LG, Pires AV, Amarante AFT (2007). Field study on nematode resistance in Nelore-breed cattle. Vet. Parasitol..

[25] Oliveira MCM, Oliveira-Sequeira TCG, Regitano LCA, Alencar MM, Néo TA, Silva AM, Oliveira HN (2008). Detection of *Babesia bigemina* in cattle of different genetic groups and in *Rhipicephalus (Boophilus) microplus* tick. Vet. Parasitol..

[26] Silva AM, Alencar MM, Regitano LCA, Oliveira MCS, Barioni Júnior W (2007). Artificial infestation of *Boophilus microplus* in beef cattle heifers of four genetic groups. Genet. Mol. Biol..

[27] Silva AM, Alencar MM, Regitano LCA, Oliveira MCS (2010). Infestação natural de fêmeas bovinas de corte por ectoparasitas na Região Sudeste do Brasil. R. Bras. Zootec..

[28] Ibelli AMG, Ribeiro ARB, Giglioti R, Regitano LCA, Alencar MM, Chagas ACS, Paço AL, Oliveira HN, Duarte JMS, Oliveira MCS (2012). Resistance of cattle of various genetic groups to the tick Rhipicephalus microplus and the relationship with coat traits. Vet. Parasitol..

[29] Medeiros MA, Barros ATM, Riet-Correa F, Marques AR, Lopes JRG, Vieira VD, Miraballes C (2019). Identification of Sindhi cows that are susceptible or resistant to *Haematobia irritans*. Rev. Bras. Parasitol. Vet..

[30] Lima LGF, Prado ÂP, Perri SHV (2002). Localização preferencial e índices diferenciados de infestação da mosca-dos-chifres (*Haematobia irritans*) em bovinos da raça Nelore. Pesq. Vet. Bras..

[31] Wang L, Sun HZ, Guan LL, Liu JX (2019). Relationship of blood DNA methylation rate and milk performance in dairy cows. J. Dairy Sci..

[32] Emam M, Livernois A, Paibomesai M, Atalla H, Mallard B (2019). Genetic and epigenetic regulation of immune response and resistance to infectious diseases in domestic ruminants. Vet. Clin.: Food Anim. Pract..

[33] Fingerman IM, Zhang X, Ratzat W, Husain N, Cohen RF, Schuler GD (2013). NCBI epigenomics: What’s new for 2013. Nucleic Acids Res..

[34] Jin L, Jiang Z, Xia Y, Lou P, Chen L (2014). Genome-wide DNA methylation changes in skeletal muscle between young and middle-aged pigs. BMC Genom..

[35] Huang YZ, Sun JJ, Zhang LZ, Li CJ, Womack JE, Li ZJ, Lan XY, Lei CZ, Zhang CL, Zhao X, Chen H (2014). Genome-wide DNA methylation profiles and their relationships with mRNA and the microRNA transcriptome in bovine muscle tissue (*Bos taurine*). Sci. Rep..

[36] Osorio JS, Jacometo CB, Zhou Z, Luchini D, Cardoso FC, Loor JJ (2016). Hepatic global DNA and peroxisome proliferator-activated receptor alpha promoter methylation are altered in peripartal dairy cows fed rumen-protected methionine. J. Dairy Sci..

